# Supplementary material for: Mechanisms governing the pioneering and redistribution capabilities of the non-classical pioneer PU.1
Source: Nat Commun. 2020 Jan 21;11:402. doi: 10.1038/s41467-019-13960-2 (PMC6972792; doi:10.1038/s41467-019-13960-2)
Supplement: Supplementary file 7 — Source data [file 41467_2019_13960_MOESM7_ESM.zip › Source_Data/FigureS3/FigureS3H_MotifScanOutput/homerResults/motif20.similar.html]

motif20

## Information for motif20

G
A
T
C
G
A
T
C
A
G
T
C
G
A
T
C
G
A
T
C
C
G
A
T
A
T
G
C
G
A
T
C
G
A
T
C
G
A
T
C
G
A
T
C
G
A
T
C
A
G
T
C
A
G
T
C
  
Reverse Opposite:  

T
C
A
G
T
C
A
G
C
T
A
G
C
T
A
G
C
T
A
G
C
T
A
G
C
T
A
G
T
A
C
G
C
G
T
A
C
T
A
G
C
T
A
G
T
C
A
G
C
T
A
G
C
T
A
G
  

|  |  |
| --- | --- |
| p-value: | 1e-39 |
| log p-value: | -9.069e+01 |
| Information Content per bp: | 1.552 |
| Number of Target Sequences with motif | 554.0 |
| Percentage of Target Sequences with motif | 14.33% |
| Number of Background Sequences with motif | 3456.5 |
| Percentage of Background Sequences with motif | 7.72% |
| Average Position of motif in Targets | 368.0 +/- 346.4bp |
| Average Position of motif in Background | 208.6 +/- 128.7bp |
| Strand Bias (log2 ratio + to - strand density) | -0.1 |
| Multiplicity (# of sites on avg that occur together) | 1.13 |
| Motif File: | file (matrix) reverse opposite |

### Similar de novo motifs found

|  |  |  |  |  |  |  |  |
| --- | --- | --- | --- | --- | --- | --- | --- |
| Rank | Match Score | Redundant Motif | P-value | log P-value | % of Targets | % of Background | Motif file |
| 1 | 0.809 | G A T C G A T C G A T C G A T C G A T C G A T C A G T C G A T C G A T C G A T C G T A C T G A C G T A C | 1e-37 | -86.538436 | 19.37% | 11.80% | motif file (matrix) |
| 2 | 0.767 | C T A G C T A G C T A G C T A G C T A G T C A G C T A G C T A G T C A G T A C G | 1e-31 | -72.184994 | 23.02% | 15.46% | motif file (matrix) |
| 3 | 0.816 | A G T C A T G C G A T C G A T C G A T C A G T C G A T C G A T C G A T C G A T C G A T C | 1e-30 | -70.198513 | 20.12% | 13.11% | motif file (matrix) |
| 4 | 0.867 | C T A G C T A G C T A G C T A G C T A G C T G A C T A G T C A G C T A G C T A G C T A G T A C G | 1e-30 | -69.944159 | 19.84% | 12.88% | motif file (matrix) |
| 5 | 0.750 | C T A G T C A G T C A G T C A G C T A G C T A G C T A G T A C G | 1e-27 | -63.172667 | 19.61% | 13.01% | motif file (matrix) |
| 6 | 0.764 | A T G C G A T C A G T C G A T C A G T C G A T C A G T C G A T C G A T C | 1e-27 | -62.297698 | 17.72% | 11.47% | motif file (matrix) |
| 7 | 0.807 | A T C G C T A G A C T G A C T G A C T G C G A T A C T G | 1e-24 | -56.370728 | 23.75% | 16.92% | motif file (matrix) |
